# A Low‐Cost Alternative Technique for Eye Protection During Prone Positioning in Spinal Surgery

**DOI:** 10.1002/hsr2.70601

**Published:** 2025-04-08

**Authors:** John Emelifeonwu, Douglas Duncan, Jay J. Park, Andreas K. Demetriades

**Affiliations:** ^1^ Department of Neurosurgery Royal Infirmary of Edinburgh Edinburgh UK; ^2^ Department of Neuroanaesthesia Royal Infirmary of Edinburgh Edinburgh UK; ^3^ Department of Neurosurgery Stanford University School of Medicine Stanford California USA; ^4^ Edinburgh Medical School, Centre for Global Health, Usher Institute The University of Edinburgh Edinburgh UK

**Keywords:** eye protection, global neurosurgery, low‐cost, POVL, Spine

## Abstract

**Background:**

Although rare, anaesthetising patinets in prone position for spinal surgery carries a risk of serious complications. Among these, postoperative visual loss (POVL) is of significant concern. Preventing POVL requires close collaboration between spinal surgeons and anaesthetists.

**Methods:**

In our centre, we practiced a cost‐effective method to ensure proper patient positioning, eye protection, and consistent assessment of eye position during prone spinal surgery. An affordable telescopic inspection mirror was used in conjunction with standard protective eye padding secured with sleek tape. This approach facilitated regular intraoperative eye checks without disrupting the surgical workflow.

**Results:**

The proposed approach offers an affordable and practical alternative to expensive commercial headrest options while maintaining effectiveness in reducing the risk of POVL.

**Conclusion:**

The method provides a viable, low‐cost solution for mitigating POVL risk in prone‐anaesthetised spinal surgery patients, highlighting the importance of interdisciplinary coordination and continuous monitoring.

## 1 Research Communication

There are numerous physiological considerations when anaesthetizing a patient in the prone position [1]. Of particular concern is the rare, unexpected, and devastating complication of postoperative visual loss (POVL), which occurs in 0.03%–0.2% of spine surgeries [2, 3]. However, given the increasing volume of spinal procedures, such devastating complications should be monitored with greater scrutiny.

The pathophysiology of POVL can be primarily divided into four categories: (i) Central Retinal Artery Occlusion (CRAO), (ii) Ischemic Optic Neuropathy (ION), (iii) Cortical blindness, and (iv) Corneal abrasion [2, 4]. ION seems to be the primary cause of POVL, but some cases are caused by CRAO. Both pathologies are mainly caused by the increase in intraocular pressure from the prone positioning [3]. Therefore, collaborative efforts between both spinal surgeons and anesthetists to optimize head positioning and reduce external compression of the eyes during surgery are imperative for preventing POVL [5].

Several risk factors contribute to the incidence of POVL, including prolonged surgical time, substantial blood loss, hypotension, and patient‐specific factors such as hypertension, diabetes, and vascular diseases. Additionally, intraoperative factors like the duration of surgery, the amount of fluid administration, and the specific type of headrest used can significantly impact intraocular pressure.

There is an increasing number of commercially available headrest options; however, these are costly. A few examples include the CS Prone Plus Head Support System, which costs £595, and the Headrest—ProneView from Mizuho OSI, which costs $1500 online. The use of these advanced headrests has been shown to reduce the incidence of POVL by optimizing head positioning and reducing ocular pressure, but their high cost can be prohibitive for many medical centers, particularly those in low‐resource settings. Foam‐based proning pillows offer a more affordable alternative, yet their prices range from ~$140 (i.e., Medline Single Use Disposable Foam Adult Head Positioner) to $270 (i.e., Mizuho GentleTouch Headrest Pillow). Given that the GDP per capita in low‐ and middle‐income countries (LMICs) ranges from $1146 to $4515, these prices still represent a significant financial burden for such settings.

**Figure 1:**
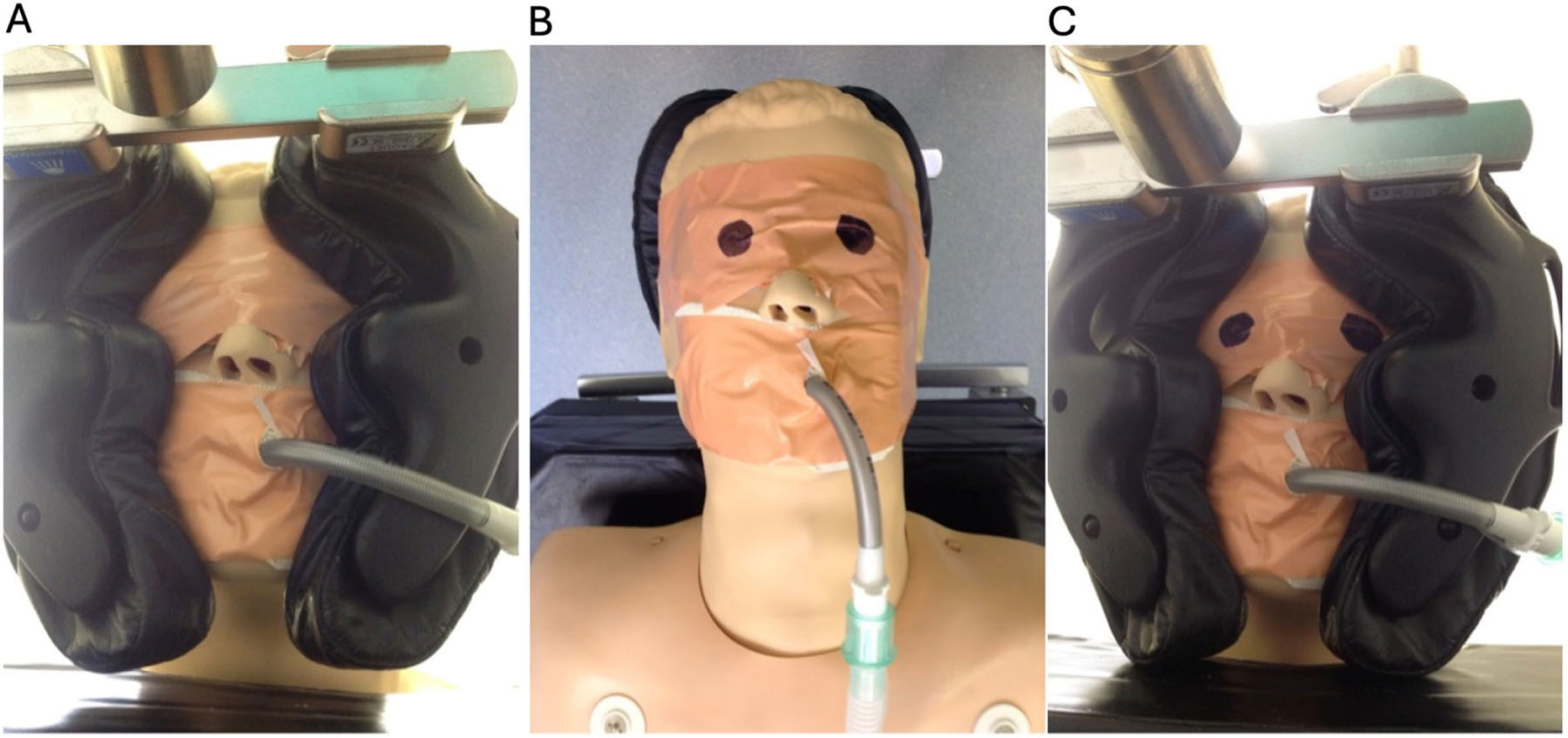
(A) Eyes obscured and position uncertain by visual inspection. (B) Position of eyes marked with marker pen before the patient is turned. (C) Eye position clearly visible in prone position.

Therefore, we aim to communicate a budget‐friendly alternative method to prevent POVL in spine surgeries. Positioning involves using an appropriate headrest, and some are more likely than others to cause ocular harm [6]. Confirmation of globe position then relies on palpation of bony landmarks (Figure 1A), while the patient is draped, risking injury to the eyes or inadvertent extubation. It is recommended that the eyes are properly positioned and checked intermittently by palpation or visualization at least every 20 min [5].

**Figure 2:**
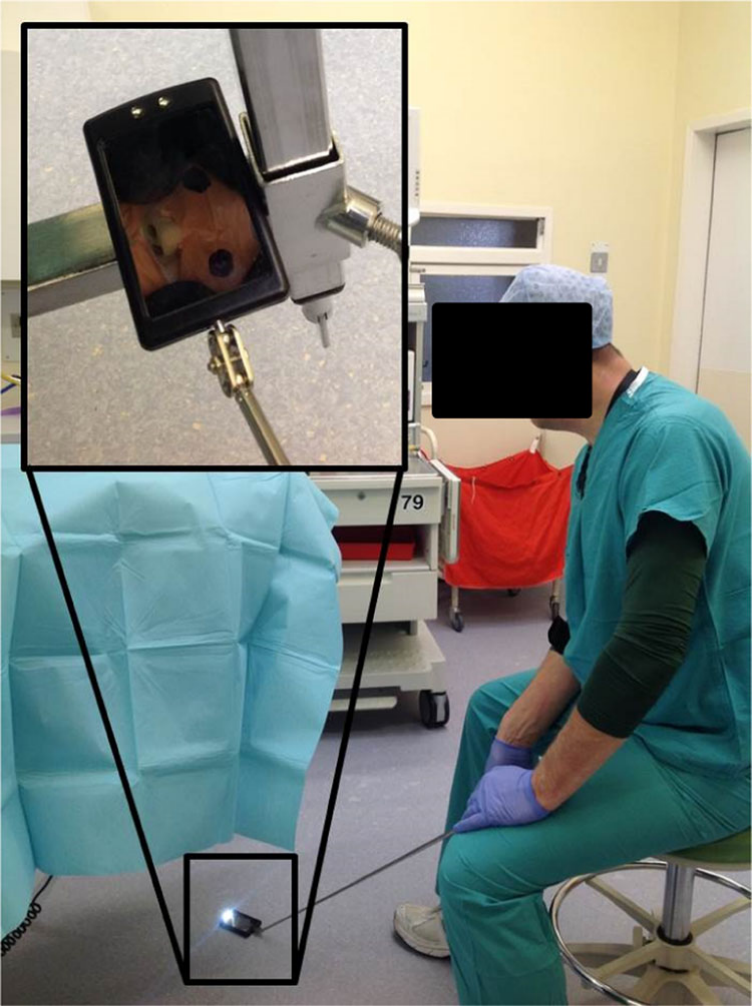
The use of a telescopic mirror allows easy conformation of eye position throughout surgery. Consent was given from the physician in the figure.

Such practice involves standard protective eye padding with a sensitive eye cover and a nonwoven eye pad, secured with sleek tape; marking of the eyeball; and intraoperative monitoring with a low‐cost mirror (< £5). We have found that marking the padded eyes with a dot before the patient is turned (Figure 1B) makes for easier appreciation of globe position when the patient is prone (Figure 1C) in comparison to Figure 1A. When combined with the use of a telescopic inspection mirror (£8 from https://Amazon.co.uk), this simple practice allows an easy and rapid assessment of eye position, ensuring continuous intraoperative protection (Figure 2). The total preparation time is < 5 min, at a total cost of under £13.

This technique provides several advantages beyond cost savings. First, it reduces the need for frequent and potentially disruptive manual palpation or visual checks of the eyes during surgery. Second, it minimizes the risk of inadvertent extubation or other injuries that could occur during manual checks. Lastly, it is easy to implement and does not require specialized training, making it accessible for a wide range of surgical teams. We present this simple and cost‐effective intervention that can be adapted for use with most of the commercially available prone positioning devices or headrests. Further prospective studies can confirm the clinical value of using this technique with minimal financial burden. We recommend this methodology especially in low‐resource areas.

## Author Contributions

**John Emelifeonwu:** conceptualization, writing – original draft, writing – review and editing, methodology. **Douglas Duncan:** conceptualization, methodology, writing – original draft, writing – review and editing. **Jay J. Park:** methodology, writing – original draft, writing – review and editing, project administration. **Andreas K. Demetriades:** conceptualization, methodology, supervision, project administration, writing – review and editing, writing – original draft.

## Ethics Statement

The authors have nothing to report.

## Consent

The authors have nothing to report.

## Conflicts of Interest

The authors declare no conflicts of interest.

## Transparency Statement

The lead author Jay J. Park affirms that this manuscript is an honest, accurate, and transparent account of the study being reported; that no important aspects of the study have been omitted; and that any discrepancies from the study as planned (and, if relevant, registered) have been explained.

## Data Availability

Any data that is included in this article are available upon the request to the corresponding author, in accordance to the Transparency and Openness Promotion (TOP) guidelines.
